# Catalytic activity of noble metals for metal-assisted chemical etching of silicon

**DOI:** 10.1186/1556-276X-7-352

**Published:** 2012-06-27

**Authors:** Shinji Yae, Yuma Morii, Naoki Fukumuro, Hitoshi Matsuda

**Affiliations:** 1 Department of Materials Science and Chemistry, Graduate School of Engineering, University of Hyogo, 2167 Shosha, Himeji, Hyogo, 671-2280, Japan

**Keywords:** Porous silicon, Metal nanoparticles, Etching of semiconductor, Displacement deposition, Electroless plating, Electrochemical catalysts, Overpotential, Galvanic etching, Oxygen reduction, Anodic dissolution of silicon, 81.05.Rm; 82.45.Vp; 81.65.Cf

## Abstract

Metal-assisted chemical etching of silicon is an electroless method that can produce porous silicon by immersing metal-modified silicon in a hydrofluoric acid solution without electrical bias. We have been studying the metal-assisted hydrofluoric acid etching of silicon using dissolved oxygen as an oxidizing agent. Three major factors control the etching reaction and the porous silicon structure: photoillumination during etching, oxidizing agents, and metal particles. In this study, the influence of noble metal particles, silver, gold, platinum, and rhodium, on this etching is investigated under dark conditions: the absence of photogenerated charges in the silicon. The silicon dissolution is localized under the particles, and nanopores are formed whose diameters resemble the size of the metal nanoparticles. The etching rate of the silicon and the catalytic activity of the metals for the cathodic reduction of oxygen in the hydrofluoric acid solution increase in the order of silver, gold, platinum, and rhodium.

## Background

Metal-assisted chemical etching of silicon (Si) has attracted considerable attention as a new electroless method that can produce porous Si by immersing metal-modified Si in a hydrofluoric acid (HF) solution without electrical bias [1-5]. Such etching generally uses not only metal-modified Si but also an oxidizing agent. The common oxidizing agent is hydrogen peroxide [1,2,4,5]. Several applications of this etching have been proposed, such as the surface-texturization (antireflection) of Si photovoltaics [6,7] and the production of Si-nanowires [8] and Si-hole arrays [9]. We have been studying the metal-assisted HF etching of Si using dissolved oxygen (O_2_) as an oxidizing agent and/or photoillumination onto Si for charge generation to promote etching [3,10]. We reported that the structure of the produced porous Si can be controlled by changing the photoillumination intensity during etching, the dissolved oxygen concentration in the HF solution, and the size and distribution of metal particles on Si [3,10-13]. This etching can be applied to the antireflection of Si photovoltaics [3,10,13-15], solar-to-chemical conversion using photoelectrochemical solar cells [15], metal nanorod formation [16], and the metallization of Si surfaces [17].

**Figure 1:**
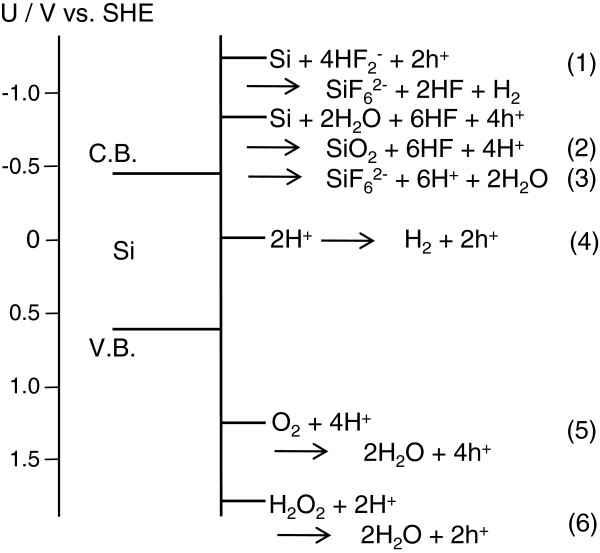
Schematic diagram of n-Si potential in a HF solution: conduction band (C.B.) and valence band (V.B.).

Metal-assisted HF etching proceeds by a local galvanic cell mechanism. Figure 1 shows a schematic diagram of the n-Si potential in a HF solution [12,18-21]. The local galvanic cell consists of the local anodic dissolution of Si (Equations 1 to 3 in Figure 1) and the local cathodic reduction on the metal (Equations 4 to 6). Three major factors control the etching reaction. The first is the photoillumination onto the Si wafers in the HF solution. It promotes etching with photogenerated charges. The photogenerated holes in the valence band of Si can etch the whole illuminated surface of the Si, and the photogenerated electrons can reduce both the oxidizing agents and protons (H^+^) on the metal particles. The second control factor is the oxidizing agent. Positive holes are injected into the valence band of the Si by suitable oxidizing agents such as oxygen and hydrogen peroxide. This process is the local cathodic reaction of the etching, which is enhanced by the oxidizing agent. Without an oxidizing agent, strong photoillumination onto Si is required to promote the etching by reducing the protons as the local cathodic reaction. The third factor is the metal particle, which is the catalyst that enhances etching. Since it localizes the etching in its vicinity under dark conditions, it can control the size of the produced Si pores. Recently, we found that palladium exhibits high activity in enhancing the HF etching of Si without an oxidizing agent, even under dissolved-oxygen-free and dark conditions [12]. In this study, except for palladium, we investigated the catalytic activity of noble metals for the etching of Si.

## Methods

Single-crystalline n-type Si wafers (CZ, (100), *ca.* 1 Ω cm, 0.5-mm thick, Yamanaka Semiconductor Co. Ltd., Kyoto, Japan) were washed with acetone and etched with a CP-4A solution (a mixture of HF, nitric acid, acetic acid, and water (3:5:3:22 in volume)) and then with a 7.3-M (M = mol dm^−3^) HF solution. Nanoparticles of noble metals (silver (Ag), gold (Au), platinum (Pt), and rhodium (Rh)) were deposited on the Si by electroless displacement deposition [22]. The Si wafers were immersed in a metal salt (silver nitrate (AgNO_3_), tetrachloroauric (III) acid (HAuCl_4_), hexachloroplatinic (IV) acid (H_2_PtCl_6_), potassium tetrachloroplatinate (II) (K_2_PtCl_4_), or rhodium chloride (RhCl_3_)) solution including 0.15-M HF. For metal-assisted HF etching, the metal particle-deposited n-Si wafers were immersed in a 7.3-M HF solution at 298 K of solution temperature for 24 h. The oxygen concentration of the HF solution was controlled by gas bubbling of oxygen and argon (Ar, >99.9999% purity, Grade-1, Taiyo Nippon Sanso Corp., Tokyo Japan). To avoid the influence of the photogenerated charges, the Si wafers were immersed in the HF solution under dark conditions. The rate of etching was measured by a gravimetric procedure using an analytical balance (XP2U, Mettler-Toledo International Inc., Columbus, OH, USA). Surface and cross-sectional inspections of the specimens were performed with scanning electron microscopes (SEM, JSM-7001 F, JEOL Ltd., Akishima, Tokyo, Japan). Electrochemical measurement was carried out with an electrochemical analyzer (VSP, Bio-logic Science Instruments, Claix, France) using a metal wire (0.12 mm in diameter) working electrode, a silver-silver chloride (Ag/AgCl) reference electrode, and a Pt counter electrode. The potential sweep at a rate of 20 mV s^−1^ started from the rest potential to the negative potential direction.

## Results and discussion

**Figure 2:**
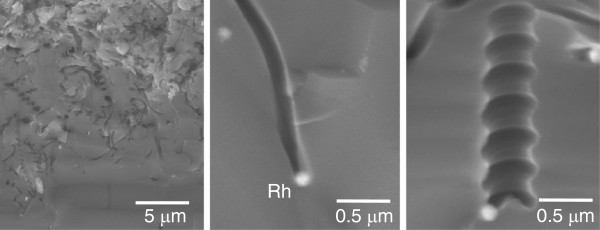
**Cross-sectional SEM images of Rh particle-deposited Si wafers after etching.** HF solution was saturated with oxygen.

**Figure 3:**
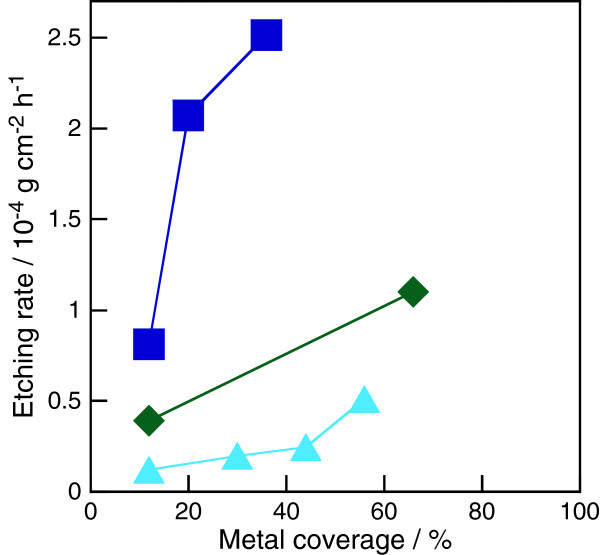
**Etching rate of metal particle-deposited Si wafers as a function of metal coverage.** HF solution was saturated with oxygen. The following marks represent the deposited metals: triangles, Ag; diamonds, Au; and squares, Pt.

Figure 2 shows the SEM images of the Rh particle-deposited n-Si wafer after being immersed in the HF solution saturated with oxygen. As the cases of the Pt [10-12], Ag [16,17], and Au [16] particles, Si nanopores were formed whose diameters resembled the size of the metal particles, which remained at the bottom of the nanopores. This result indicates that the etching reaction proceeded under the metal particles. Thus, we expect that the etching rate measured by the gravimetric procedure depends on the metal coverage of the Si surface. Figure 3 shows the etching rate as a function of the metal coverage of the Si surfaces. The coverage was determined using the percentage of white parts in the SEM images. Higher metal coverage gives a higher etching rate.

**Figure 4:**
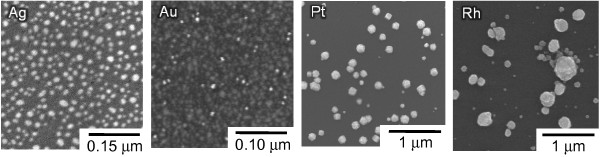
SEM images of metal particle-deposited Si wafers.

**Figure 5:**
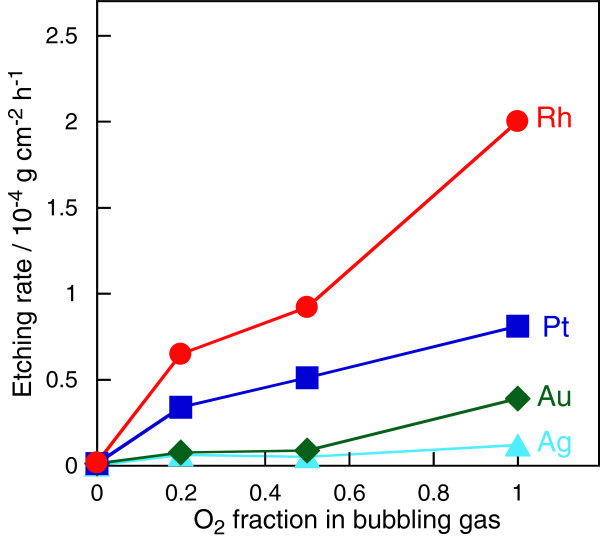
**Etching rate of metal particle-deposited Si wafers versus oxygen fraction in bubbling gas.** HF solution was bubbled with a mixture of pure argon (Ar) and pure O_2_. The following marks represent the deposited metals: triangles, Ag; diamonds, Au; squares, Pt; and circles, Rh.

To compare the catalytic activity of the metals for the metal-assisted HF etching of Si, we controlled its metal coverage by changing the metal deposition conditions. Figure 4 and Table 1 show the SEM images, the deposition conditions, the particle density, and the particle size of the metal particle-deposited n-Si wafers controlled at 12% of metal coverage. The etching rates of these Si wafers are indicated in Figure 5 for four kinds of gas bubblings: pure argon, i.e., no dissolved oxygen in the HF solution, mixtures of oxygen and argon (1:4 and 1:1 in flow rate), and pure oxygen, i.e., oxygen saturated in the HF solution. The etching rate increased with the oxygen fraction in the bubbling gas, that is, the concentration of the dissolved oxygen in the HF solution and in the order of Ag, Au, Pt, and Rh. The etching rate using the oxygen-free HF solution was between 0.002 × 10^−4^ and 0.017 × 10^−4^ g cm^−2^ h^−1^, which resembles the 0.003 × 10^−4^ g cm^−2^ h^−1^ of the bare Si wafers. The rate using the oxygen-saturated HF solution was between 0.12 × 10^−4^ and 2.0 × 10^−4^ g cm^−2^ h^−1^, which is much higher than the 0.005 × 10^−4^ g cm^−2^ h^−1^ of the bare Si wafers. These results indicate that the protons in the HF solution cannot inject positive holes into Si even under the presence of such catalytic noble metals as Pt and Rh (Equation 4 in Figure 1), and neither a bare Si surface nor the four examined noble metals have catalytic activity for the oxidation of Si with water and/or fluoride species in the HF solution (Equations 1 and 2 in Figure 1) even if these reactions proceed thermodynamically. They also indicate that the four noble metals promote the positive-hole injection into the Si valence band by oxygen in the HF solution (Equation 5 in Figure 1), and thus, the dissolved oxygen can effectively work as the oxidizing agent only under the presence of catalytic metals. Therefore, the etching rate depends on the catalytic activity of the metal particles on Si for the cathodic reduction of dissolved oxygen in the HF solution.

**Table 1:** Deposition conditions, particle density, and size of metal particles on Si wafers

**Metal**	**Metal salt concentration(×10**^**−3**^ **mol dm**^**−3**^**)**	**Solution temperature (K)**	**Deposition time (s)**	**Average particle density (×10**^**11**^ **cm**^**−2**^**)**	**Average particle size (nm)**
Ag	1.0	278	5	1.8	9
Au	0.5	278	10	5.2	5
Pt	1.0	313	90	0.066	50
Rh	1.0	313	120	0.048	60

Metal salt for Pt deposition was potassium tetrachloroplatinate (II).

**Figure 6:**
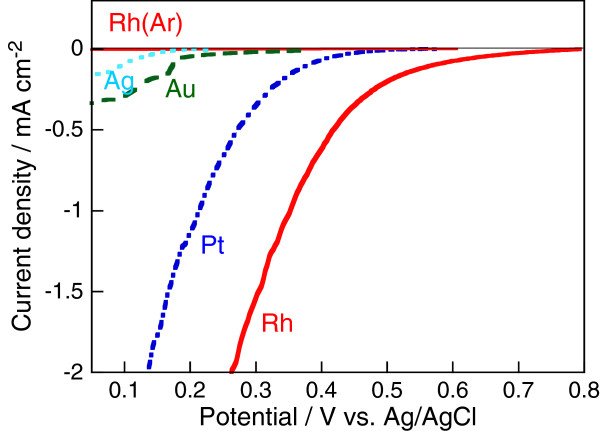
**Current density versus potential curves of metal electrodes in oxygen-saturated HF solution.** Lines indicate the following metal electrodes: dotted, Ag; dashed, Au; dot-dashed, Pt; and thick-solid, Rh. Fine-solid line indicates Rh electrode in oxygen-free HF solution (pure argon bubbling).

To examine the catalytic activity of the metals for oxygen reduction, we measured the current density versus the potential curves of the metal electrodes in the oxygen-saturated HF solution (Figure 6). The cathodic current obtained in the potential range indicated in Figure 6 is for the oxygen reduction because no cathodic current was obtained for the oxygen-free HF solution with argon bubbling as the fine solid curve for the Rh electrode in Figure 6. The on-set potential of the cathodic current shifted towards the positive direction in the same order of Ag, Au, Pt, and Rh as the etching rate of Si. Also, the potential for the current density corresponding to the etching rate of Si shifted towards the positive direction in the same order. These results indicate that the catalytic activity of the metals to the cathodic oxygen reduction in the HF solution increases, and the overpotential of the local cathodic reaction of the metal-assisted HF etching of Si decreases in the order of Ag, Au, Pt, and Rh. The decrease in the overpotential of the local cathodes positively shifts the mixed potential of the local galvanic cells and increases the current density of the galvanic cells, i.e., the etching rate of Si.

## Conclusions

In this study, we investigated the catalytic activity of noble metals, Ag, Au, Pt, and Rh, for the metal-assisted HF etching of Si using dissolved oxygen as an oxidizing agent. The catalytic activity of the noble metals for the cathodic oxygen reduction in a HF solution increases in the order of Ag, Au, Pt, and Rh. The activity controls the local cathodic reaction of the metal-assisted HF etching of Si under dark conditions. Thus, the etching rate of Si is determined by the dissolved oxygen concentration, the kind of metals, and the metal coverage of the Si surfaces.

## Abbreviations

Ag: silver; Au: gold; HF: hydrofluoric acid; O2: oxygen; Pt: platinum; Rh: rhodium; SEM: scanning electron microscope; Si: silicon.

## Competing interests

The authors declare that they have no competing interests.

## Authors’ contributions

SY conceived, designed, and coordinated the study and drafted the manuscript. YM carried out the experiments and helped draft the manuscript. NF and HM participated in the coordination of the study. All authors read and approved the final manuscript.

## Authors’ information

SY is an associate professor, YM is a graduate school student, NF is a research associate, and HM is a professor at Department of Materials Science and Chemistry, Graduate School of Engineering, University of Hyogo.
